# Adaptations to “Thermal Time” Constraints in *Papilio*: Latitudinal and Local Size Clines Differ in Response to Regional Climate Change

**DOI:** 10.3390/insects5010199

**Published:** 2014-01-21

**Authors:** J. Mark Scriber, Ben Elliot, Emily Maher, Molly McGuire, Marjie Niblack

**Affiliations:** Department of Entomology, Michigan State University, East Lansing, MI 48824, USA; E-Mails: ellio187@gmail.com (B.E.); emilymaher@gmail.com (E.M.); mollymcguire88@gmail.com (M.M.); niblackm@msu.edu (M.N.)

**Keywords:** Papilionidae, thermal landscapes, developmental constraints, introgressive hybridization, Voltinism/Size/Degree-day Model, phenotypic flexibility, Bergmann’s Rule

## Abstract

Adaptations to “thermal time” (=Degree-day) constraints on developmental rates and voltinism for North American tiger swallowtail butterflies involve most life stages, and at higher latitudes include: smaller pupae/adults; larger eggs; oviposition on most nutritious larval host plants; earlier spring adult emergences; faster larval growth and shorter molting durations at lower temperatures. Here we report on forewing sizes through 30 years for both the northern univoltine *P. canadensis* (with obligate diapause) from the Great Lakes historical hybrid zone northward to central Alaska (65° N latitude), and the multivoltine, *P. glaucus* from this hybrid zone southward to central Florida (27° N latitude). Despite recent climate warming, no increases in mean forewing lengths of *P. glaucus* were observed at any major collection location (FL to MI) from the 1980s to 2013 across this long latitudinal transect (which reflects the “converse of Bergmann’s size Rule”, with smaller females at higher latitudes). Unlike lower latitudes, the Alaska, Ontonogon, and Chippewa/Mackinac locations (for *P. canadensis*) showed no significant increases in D-day accumulations, which could explain lack of size change in these northernmost locations. As a result of 3–4 decades of empirical data from major collection sites across these latitudinal clines of North America, a general “voltinism/size/D-day” model is presented, which more closely predicts female size based on D-day accumulations, than does latitude. However, local “climatic cold pockets” in northern Michigan and Wisconsin historically appeared to exert especially strong size constraints on female forewing lengths, but forewing lengths quickly increased with local summer warming during the recent decade, especially near the warming edges of the cold pockets. Results of fine-scale analyses of these “cold pockets” are in contrast to non-significant changes for other *Papilio* populations seen across the latitudinal transect for *P. glaucus* and *P. canadensis* in general, highlighting the importance of scale in adaptations to climate change. Furthermore, we also show that rapid size increases in cold pocket *P. canadensis* females with recent summer warming are more likely to result from phenotypic plasticity than genotypic introgression from *P. glaucus*, which does increase size in late-flight hybrids and *P. appalachiensis*.

## 1. Introduction

The adaptations to severe thermal constraints on developmental rates and voltinism for high latitude populations or species of insects (such as tiger swallowtail butterflies of North America) involve most life stages and have been shown to include: (1) smaller pupae and correspondingly smaller adult sizes (earlier pupation) which largely explains a general latitudinal size cline, decreasing from Florida to Alaska [1,2]; (2) larger eggs, permitting a fast start for neonate larvae [3]; (3) oviposition preference for the most nutritious larval host plants locally that permit rapid larval growth as in the “voltinism suitability” hypothesis of Scriber and Lederhouse [4,5,6]; (4) larval and adult solar basking [7,8]; (5) a lower diapause intensity, permitting earlier spring adult emergences from post-diapause pupae [9,10,11,12]; (6) faster larval growth and molting, especially at cooler temperatures [3,8]; and (7) photoperiodically-insensitive obligate diapause, resulting in univoltinism in populations across all of Canada and the eastern USA north of the historical hybrid zone [13,14].

**Figure 1:**
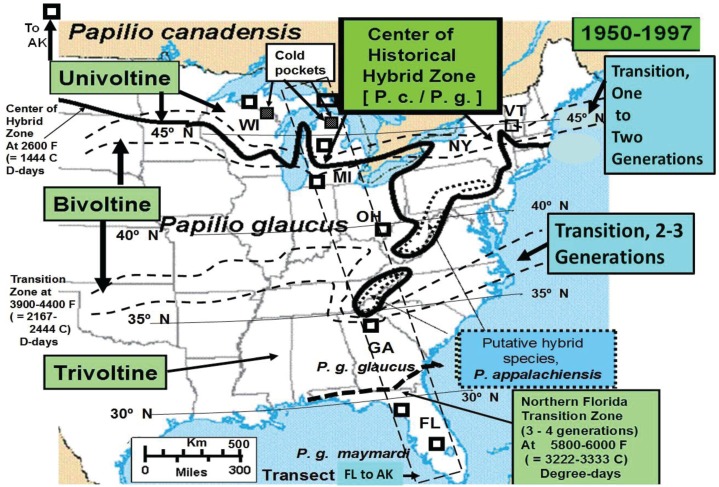
The geographic location of key sites along the latitudinal transect showing the historical center of the *P. canadensis*, *P. glaucus* hybrid zone is indicated by dark line. The northern *P. canadensis* is univoltine (obligate diapauser) while *P. glaucus* is everywhere multivoltine (facultative diapauser). The northern limits of bivoltine potential [4] turns out to be roughly at the 2,600–2,700 Degree-days F (Figure 2), not as shown here across New York State. The location of transition zones from univoltine to bivoltine populations have historically been very similar for the European corn borer moth and tiger swallowtail butterflies [4,6]. The hybrid species, *P. appalachiensis*, is found on the warmer side of the hybrid zone [1,9]. Key *P. glaucus* sites from south to north include Highlands County (=Co.), FL; Levy Co., FL; Clarke and Oglethorpe Cos., GA; Gallia, Lawrence, Adams Cos., OH; and St Joseph Co., MI. Florida populations have 3–4 generations (see Section 3.1, on Voltinism/Size/D-day Model) and have been considered a separate subspecies, *P. g. maynardi* [2].

The univoltine *Papilio canadensis* (R & J) adults appear in mid-May through late June from Alaska to the Great Lakes and New England hybrid zone (Figure 1). Along the northern edge of its range, near the hybrid zone, which is also at the transition zone between voltinism patterns at roughly 2,600 Degree-days F, base 50° F (=1444 D-days C, base 10° C), the bivoltine *P. glaucus* L. is parapatric with its univoltine sister species, *P. canadensis*, and also has adults in flight during late May and June. Further south, in Florida the *P. glaucus* has an earlier spring flight in mid-March to early April [15,16]. The spring flight of adults in Georgia to southern Ohio usually occurs in April and May. The transition zone from two-three generations is variable across latitude because of the Appalachian Mountains, but appears to be basically defined by the thermal landscape where 3,900–4,400 F D-days (=2,167–2,444 C D-days) occur during the growing season (Figure 1).

Latitudinal size clines have been documented empirically in males [2] and also in females, which are both larger at lower latitudes [17]. In order to understand why adaptive thermal responses vary temporally and geographically, even within a species, we need to understand behavioral, physiological, and ecological mechanisms involved for different genotypes among populations such as with these *Papilio* species and their hybrids. In theory, several factors may affect such latitudinal size clines, including trade-offs between size, growth rates, host plants, and voltinism [18,19,20,21,22,23,24,25]. The size of female Lepidoptera is often positively correlated with fecundity [26]. Over a 30-year period (including 10–15 years of exceptional recent warming), we have evaluated the thermal landscape as a predictor of voltinism and size trends in selected populations across latitude from Florida to Alaska. From these data we developed and present here an empirically-derived model for “Size/Voltinism/Degree-days” on the thermal landscape for the summer growing season.

Natural hybridization along this thermally-defined zone of ecological transition and hybrid interaction [26] from the Great Lakes to New England has resulted in some homoploid recombinant *Papilio* hybrids that have evolutionarily diverged from both parental species due to temporal isolation via a 2–4 week delay in their post-diapause emergences [9,27,28,29]. Time of post-diapause spring emergences from overwintering pupae may contribute significantly to this observed ecological and evolutionary separation among these three *Papilio* species (*P*. c., *P*. g., and *P*. a.) and their “late-flight” hybrid swarms across the hybrid zone [27]. Delayed “late flight” hybrid adult flight times provide immediate temporal separation from both parental species in and around the hybrid zone (e.g., at higher altitudes), and is a significant reproductive isolation mechanism that certainly helped in generating the hybrid species of Mountain Swallowtail butterfly (*P. appalachiensis*) in thermally-defined areas along the univoltine/bivoltine transition zone ([1,28,29,30]; see also Figure 1).

The ecological importance of voltinism differences for evolutionary divergence and the insipient speciation process cannot be overemphasized [1]. Climate induced genetic introgression may provide important contributions to preserving “cryptic” biodiversity [31,32,33] and could enhance understanding of community-level differences in rates of range change responses to climate warming. We present data for the latitudinal clines in female forewing lengths, updated from 1992 [17], through 2013, for selected populations from Florida (27° N latitude) to Alaska (65° N latitude). Means for these tiger swallowtail butterfly populations were each evaluated for potential size increases during the past two-three decades where annual mean summer D-day accumulations have increased significantly [1]. Since significant recent winter warming had been documented around the “climatic cold pockets” of northern Michigan (two full plant hardiness zone shifts from 1990 to 2006; see Section 2.3), additional population samples and D-day analyses were made there, at a finer geographic scale. Females around these local “cold pockets” would have been under severe thermal constraints on their size for previous decades from both colder winters and colder summers (1950–1990). For example, in addition to lower summer D-day totals, they had a mean of only 70–80 days between the last spring freeze and the first fall freeze (and their forewings averaged only 41–45 mm) compared to locally adjacent warmer areas with 100–110 days between these Spring-Fall freeze dates (where forewing lengths were 46–50 mm [17]).

## 2. Experimental Section

### 2.1. Developmental Thresholds for Larval Growth

**Figure 2:**
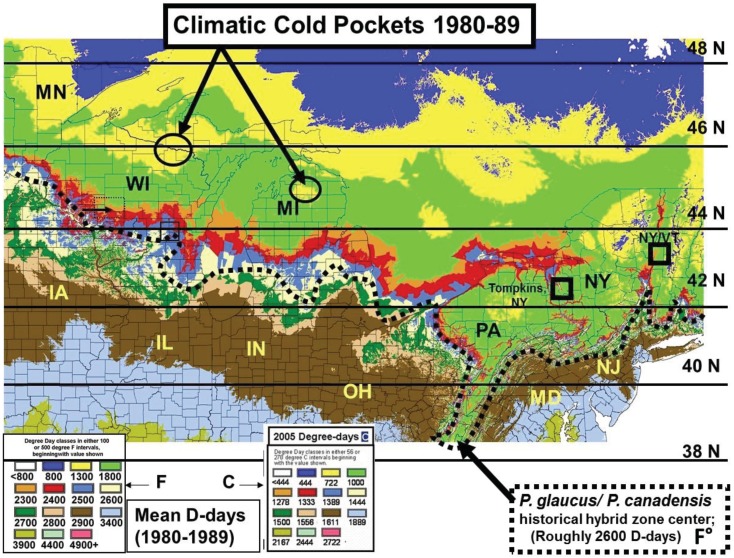
The 10-year average thermal unit accumulations (above 50° F = 10° C) across the northeastern USA for 1980–1989. The 50-year historical hybrid zone (1950–1999) is indicated by Degree-day totals from 2300–2900 F (or 1278–1611 C).

**Figure 3:**
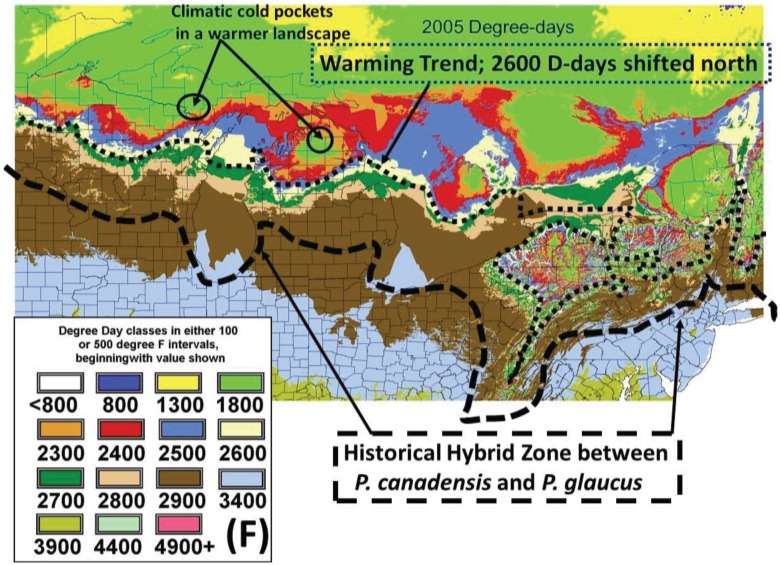
(Summer Warming) Recent warming as shown here for 2005, shows an extensive shift northwards (e.g., dark brown color = 2900–3400 D-days F), where all populations are potentially bivoltine (even on poor host plants). This rapid warming fills or exceeds the entire historic hybrid zone, apparently driving recent genetic introgression and some (but not all) species diagnostic traits extensively northward (with 500–800 D-day increases locally across the thermal landscapes into higher latitudes and altitudes).

**Figure 4:**
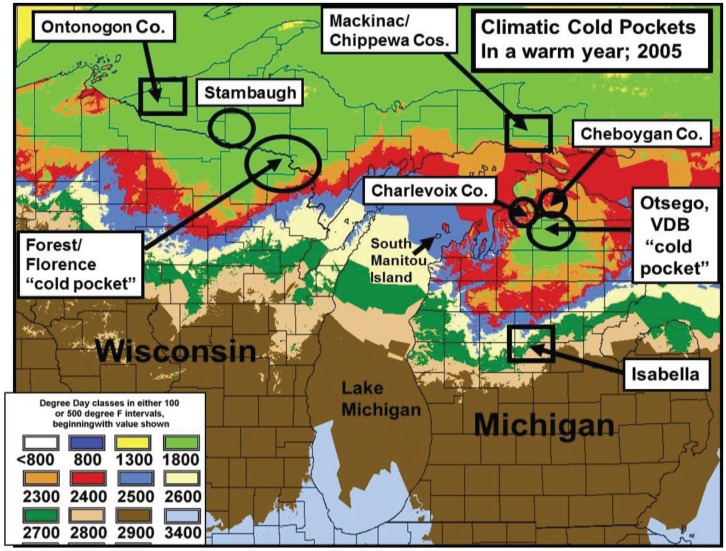
The thermal landscape (fine-scale) showing warming in the year 2005 near the “climatic cold pockets” of Michigan (Otsego Co., MI, USA) and Wisconsin (Forest/Forest Cos.). (compare Figure 2 from 1980–1989).

The lowest temperature for development (base threshold) is used in growing Degree-day calculations [34]. Degree-days have been especially valuable for modeling potential population growth and predicting when various phenological events might be expected from year to year (more effectively than calendar date) [35]. The base developmental threshold was calculated as the inverse of the time required to complete development when plotted against rearing temperatures. The larval growing degree-days for *Papilio* larvae had been previously determined to be approximately 10° C (=50° F) [36,37,38,39] and this basal developmental threshold was used to develop the thermal landscape maps (annual mean Degree-days) for this paper (e.g., Figure 1, Figure 2, Figure 3 and Figure 4). The degree-day calculations done by Zedex Inc. (Belefonte, PA, USA) were daily mean temperatures, with no upper thermal cut-offs (as with corn growth at 86° F). The upper growth thresholds for *Papilio* are not known.

### 2.2. Calculation of Local Growing Degree-Days and Seasonal “Thermal Landscape” Mapping

The daily thermal unit accumulations through the growing season March 1st–October 31st (in Fahrenheit degree-days above a base 50, =9/5 C Degree-days above a base 10° C) were calculated annually for each of more than 2,000 stations in northeastern USA (by Zedex, Inc, Bellfonte, PA, USA [35]). These historical degree day accumulations were run in a GIS spatial program incorporating influences of altitude as well as latitude with interpolation to 1 km² and presented with color coding of “thermal distances” to reflect isotherms at 100 degree-day intervals between 2,300–2,900 F (and at 500 degree day intervals to the north and south of this critical thermal transition zone; see Figure 2 and Figure 3). Other climatic data were obtained from the USDA Plant Hardiness Zone maps and the Climatic Atlas of Michigan [40].

### 2.3. Cold Pocket Sampling sites in Northern Michigan and Wisconsin

**Figure 5:**
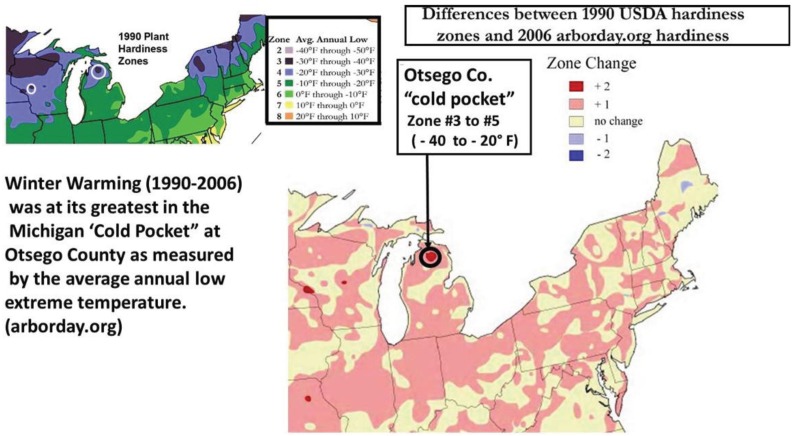
(Winter Warming) Plant Hardiness Zones are shown for Michigan, Wisconsin, and New England. These zones reflect the mean of the extreme coldest temperatures annually. In northern WI and northern MI there has been significant warming up to and beyond 2006 (lower left; averages for these zones are calculated including the entire period of the 1974–1986 data, as well as the 1986–2005 data from the warmer period). Nonetheless, the Michigan cold pocket has experienced two Zone shifts for Hardiness Zones since the 1990 version in the upper left; USDA).

Climatic cold pockets had been described by Scriber earlier [17] as a likely strong selection force resulting in smaller females locally for *P. canadensis*. Here we updated the butterfly population sampling (and Degree-day accumulation totals per year) from 1993 to include potential forewing changes through the recent 10–15 years of exceptional general climate warming (Figure 2 and Figure 3). In addition to the latitudinal macro-analysis population sites of Ontonogon, Mackinac/Chippewa Cos. in the Upper Peninsula (UP), and Isabella Co. in central MI (Lower Peninsula; indicated by squares in Figure 1 and Figure 4), we examined cold pocket populations at a finer level in the Otsego cold pocket area in Michigan’s lower peninsula and the Forest and Florence counties (near Iron Mt.) in Wisconsin (Figure 4). Adjacent populations were also examined near Otsego (Charlevoix and Cheboygan Cos.) and in the Upper Peninsula further into the UP cold pocket (Stambaugh, in Iron Co., MI, USA). Plant Hardiness Zone warming has occurred across North America during the past 10–15 years, with the greatest increase east of the Mississippi River (a two-zone shift) occurring in the Otsego County, Michigan “cold pocket”. Here, the coldest extreme cold temperatures have changed from −40° F (in 1990) to −20° F (in 2006; Figure 5).

### 2.4. Insects

Adult *Papilio* specimens were field-collected at key sites along a transect from southern Florida (27° N latitude) to Alaska (65° N) from 1980–2013 (Figure 1). Forewing lengths of females were measured (to the nearest mm) for this study. The historical patterns of voltinism (at transition zones) in tiger swallowtai1 butterflies *(Papilio*) [4,41,42] and, incidentally also for the European corn borer (*Ostrinia*) [43,44] from 1950–1990 are shown geographically (Figure 1). Over a 30-year period (1984–2013), the pattern in mean female forewing lengths of *Papilio* (including the univoltine *P. canadensis*, the “late flight” recombinant hybrid populations, and *P. appalachiensis*, as well as the FW size of bivoltine/multivoltine flights of *P. glaucus*) are shown in relation to the thermal landscape (mean annual Degree-day accumulations, March 1st–Oct 31st) in an empirically-derived Voltinism/Size/D-day model.

**Figure 6:**
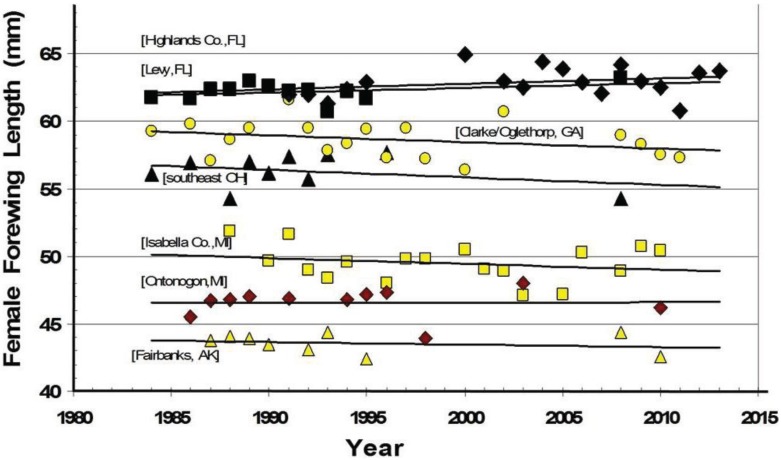
The mean annual forewing lengths of females collected at key locations across the latitudinal transect from Florida to Alaska for the three recent decades. The sample sizes, R-square values for trend lines, and the mean of the annual means (shown in Figure 2) are presented in Table 1. Highlands Co. and Levy Co. FL have very similar trend lines.

**Figure 7:**
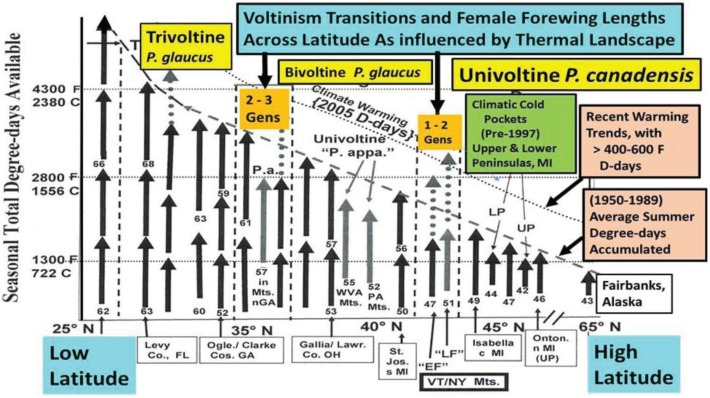
An empirically-derived Model of the latitudinal trends in female forewing size and voltinism as a function of “thermal time” (Degree-day accumulations; mean from 1950–1989) from Florida at 27° N to Alaska at 65° N latitude (developmental base 50° F; =10° C). Actual observed size differences (see Table 1) of swallowtail butterfly female forewings (FW) are roughly indicated by the arrow lengths, and show a general cline of decreasing size with increasing latitude (Converse Bergmann’s Rule). In the past decade, increases of roughly 700 D-days F (=400 D-days C; see Figure 3) are represented by the relaxation (upward shift indicated) of the constraints (dotted line) [l]. Selected sites along this 3,500 km latitudinal transect are depicted annually (Figure 1), show consistency of wing lengths during the past 4 decades (Figure 6) for *P. glaucus* (>2,500 females) and the northernmost *P. canadensis* (>1,000 females). Note the univoltine “LF”hybrids and “EF” *P. canadensis* at 43° N latitude, and also the univoltine *P. appalachiensis* populations in Munroe Co. PA (40° N) and Pendleton Co. West Virginia (39° N) and Rabun & Habersham Cos. GA (36° N). These localized mountain populations have thermal constraints much greater than surrounding populations (as with the Michigan cold pockets, which realize considerably fewer thermal units locally than shown by the general 1950–1989 dotted line).

Forewing length data from various years (including updates from the most recent 2 very warm decades) for these populations are presented below (in Figure 6 and Table 1). These data, from key long-term sampling sites served as the basis for the empirically-derived size/voltinism/thermal landscape model presented below (Figure 7). Data for the Chippewa/Mackinac Cos. and are not presented graphically (below) due to space constraints (and also due to Ontonogon site at the same latitude), but no significant changes in forewing length through time were present its trend lines (see Figure 6, Table 1).

## 3. Results

### 3.1. The Historical Thermal Landscape of Eastern North America and a “Voltinism/Size/D-day” Model

The thermal landscapes based on seasonal D-day totals (or growing degree-days; base thresholds of 50° F = 10° C) nicely delineate the plant and insect transition zones for many hybridizing species [4,26], and have shaped (constrained) the voltinism patterns of *P. glaucus* and, incidentally in a very similar way as seen for *Ostrinia* (corn borer moth ecotypes) across eastern North America (Figure 1; [35,38,43]). Temperature is the main driving factor in insect development (for any given nutritional quality of host plant [22,23,24]) and D-days help predict their growing season “time” (=developmental) constraints. The basic voltinism of *P. glaucus* (and *P. canadensis* and *P. appalachiensis*) is described (Figure 1) from outhern FL to central AK in an empirically-based “voltinism/size/D-day model” based on field data collected at selected sites across this area (below) over a 3-decade period from 1970–1997 (D-days means very similar to the 1980–1989 decade, Figure 2). The recent 15 years (1998–2012) have been significantly warmer across eastern North America with annual temperature constraints relaxed by several hundred D-days [1], as shown in Figure 3 (with 2005 as a warm year). However, despite significant climate warming during the past 15 years, with several hundred degree­day increases near the hybrid zone (Figure 3), the general wing lengths of females across this latitudinal step cline have not varied much at any of our long-term sampling sites (Figure 6; Table 1).

The historical latitudinal transitional junctures (voltinism transition zones, from 1–2 and 2–3 generations) for tiger swallowtail butterflies are shown with the associated Degree-days generally required on Y-axis (Figure 7). The increased number of growing days for immature insects (Seasonal total Degree­days) in lower latitudes allows a longer time for development and therefore bigger individuals (the “Converse of Bergmann’s Rule” [19]). This is seen for *P. glaucus* in Florida 27–29° N latitude, where the female forewing length s average 63 mm in the spring and 68 mm in the late Summer/Fall, compared to smaller sizes northward into northern GA, southern OH, southern MI. Late Summer flights in multivoltine populations are bigger than the Spring individuals (Figure 7; Table 1). Sizes at these latitudinal macrosites (Figure 1) are surprisingly stable in the face of significant climate warming, possibly due to trade-offs of unknown nature [18,19]. This macro-latitudinal size decline continues for the univoltine *P. canadensis* with 48 mm in central MI (at 42° N latitude.) dropping to 40–42 mm in AK at 65° N latitude (but also locally at some lower latitudes; 42–43 mm in northern MI climatic “cold pockets”; Figure 7 [5,17]).

**Table 1:** The mean (of annual means) of Forewing lengths (mm) of yellow females of *P. glaucus*, *P. canadensis*, at selected sites from Florida (27° N latitude) to Alaska (65° N latitude; see Figure 1). Vermont late-(LF) hybrids and early flight (EF) are included for comparison. Correlation coefficients show no relationships between FW length and year for any *P. glaucus* population or at Alaska, Ontonogon, and Mackinac Cos. (Figure 6). In contrast, “cold pocket” populations in MI and WI (at the bottom) showed significant increases in forewing lengths (see also Figure 8, Figure 9, Figure 10 and Figure 11). (Significant correlations *** *p* = 0.001, ** *p* = 0.01). NA = not sampled.

		Spring Flight				Late Summer			
County, State (1984–2013)	Degrees N latitude	Mean Forewing Length mm)	Yrs (n)	Total Females (n)	R² Spring Flight	R² Summer Flight	Mean Forewing length (mm)	Yrs (n)	Total Females (n)
*P. glaucus* Highlands, southern FL	27° 28'	62.3	10	337	0.10 n.s.		66.0	2	14
Levy, north FL	29° 12'	62.9	18	530	0.06 n.s.		68.5	2	13
Clarke Co. north GA	33° 56'	52.1	3	184	NA	0.07 n.s.	58.5	17	492
Southeast OH	38° 40'	51.7	2	14	NA	0.05 n.s.	56.3	8	387
St. Joseph, MI (Pg)	41° 52'	50.3	2	6	NA	0.15 n.s.	54.6	6	47
*P. canadensis* Isabella, central MI	43° 48'	49.5	18	241	0.05 n.s.	Univoltine			
Mackinac, MI (UP)	46° 10'	46.9	12	96	0.06 n.s.	Univoltine			
Ontonogon, MI (UP)	46° 30'	46.7	10	127	0.0003 n.s.	Univoltine			
Fairbanks, AK	64° 55'	43.6	9	163	0.05 n.s.	Univoltine			
Battenkill River, VT “EF”(Pc)	43° 6'	48.3 ± 1.1	10	378	0.007 n.s.	Univoltine early(May–June)	Hybrid Zone (VT)		
Battenkill River “LF” Hybrids	43° 6'	50.9 ± 1.5	8	190	0.021 n.s.	Univoltine Late (July)	Hybrid Zone (VT)		
Cold pocket, Otsego, MI	45° 8'	49.5 ± 0.9	13	125	0.57***	Univoltine	Figure 8		
Charlevoix Co. near “CP”	45° 11'	48.0 ± 1.0	18	309	0.46 ***	Univoltine	Figure 9		
Cheboygan Co. near “CP”	45° 22'	48.9 ± 0.8	13	145	0.23 **	Univoltine	Figure 10		
Cold Pocket Florence & Forest, WI	45° 56'	45.6 ± 2.4	8	34	0.50 ***	Univoltine	Figure 11		

### 3.2. In Contrast, Females in “Cold Pocket” Populations do Show Increased Size with Recent Warming

**Figure 8:**
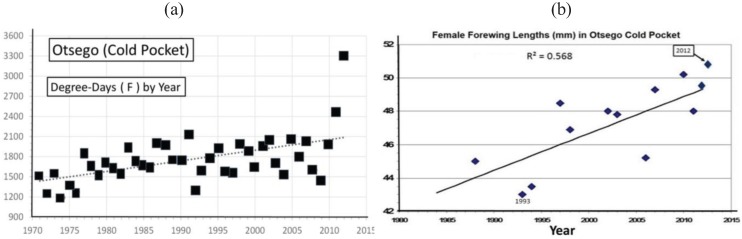
(**a**) The summer degree-day increase in the Michigan “cold pocket” at Otsego County; and (**b**) The female *P. canadensis* forewing increase in this Otsego Co. cold pocket (see Table 1).

**Figure 9:**
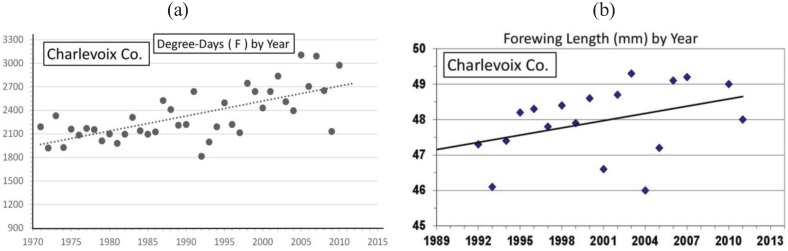
(**a**) The summer degree-day increases; (**b**) female *P. canadensis* forewing increase in Charlevoix Co. (adjacent to Otsego Co.; see Figure 4 and Table 1).

**Figure 10:**
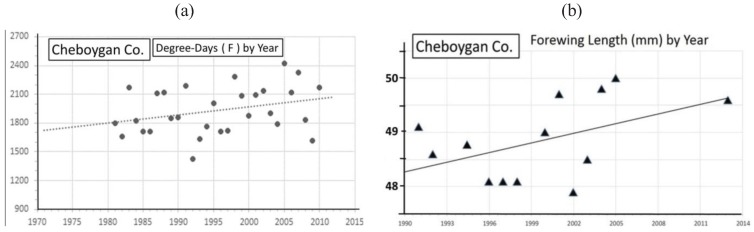
(**a**) The summer degree-day increases; (**b**) female *P. canadensis* forewing increase in Cheboygan Co. (adjacent and north of Otsego Co.; see Figure 4 and Table 1).

**Figure 11:**
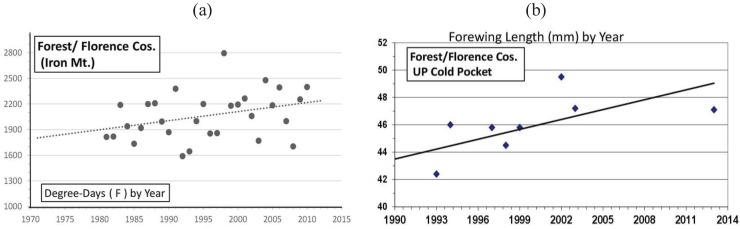
**(a**) The summer degree-day increases in the Wisconsin “cold pocket” at Forest/Florence Cos.; (**b**) the female *P. canadensis* forewing increases in this cold pocket (Table 1).

**Figure 12:**
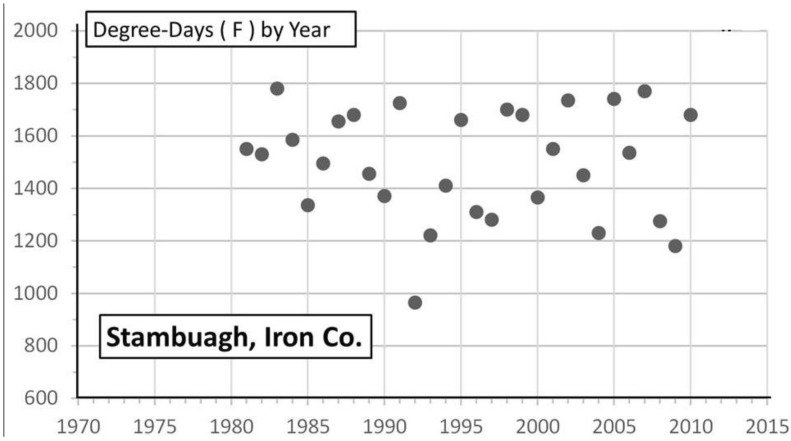
The lack of degree-day increases over 30 years in Iron Co. at Stambaugh (which is slightly north of the cold pocket center; R² = 0.011). A similar lack of warming was seen at Ontonogon, and Mackinac/Chippewa Cos. (see Figure 4).

Significant warming in the northern Michigan cold pocket at Otsego Co. has occurred and may have been a major factor that generated the significant annual female size increases during the past 2 decades (Figure 8a,b). Also at the adjacent counties of Charlevoix and Cheboygan (see Figure 4), similar increases in summer degree-days (Figure 9a and Figure 10a) and female forewing sizes were also observed (Figure 9b and Figure 10b). An increase in annual mean forewing sizes in the northern Wisconsin Forest/Florence Cos. cold pocket was also observed, along with significant summer warming at the eastern side Iron Mountain population (Figure 11a,b). However, at the center of the Upper Peninsula cold pocket, significant summer warming did not occur (Stambaugh, Iron Co. MI, USA; Figure 12). Such a lack of significant warming during the past 15 years was also the case for the Ontonagon and Mackinac/Chippewa sites in the Upper Peninsula discussed earlier in latitudinal cline study, and no increase in forewing size of *P. canadensis* was seen (Table 1)

## 4. Discussion

Evidence of recent climate change impacts on butterfly ranges and hybridization is extensive [1,45,46,47,48,49,50,51,52,53,54]. Mean annual growing degree-days (above base 50° F, = 10° C) across eastern North America reflect strong elevational as well as latitudinal clines in selection pressures. They are used here to illustrate the thermal landscape that shapes the voltinism patterns of *Papilio* species of the tiger swallowtail butterflies across eastern North America (Figure 1). At our major collecting sites in Florida, Georgia, Ohio, Michigan and Alaska (from 1984 to 2013; Table 1) female forewing sizes of these butterflies reflect a basically stable multi-year latitudinal size cline, despite significant increases across the landscape in thermal units (as much as 400–700 D-days F; as seen in 2005; Figure 3; and even larger increases in 2010 and 2012) due to significant regional climate warming during the past 15 years. This clinal size pattern of smaller size at higher latitudes reflects the Converse of Bergmann’s Rule, which mostly occurs in larger insects with one (or few) generations per year compared to smaller insects with many generations per year or many years per generation [19,55,56,57]. Seasonal time (thermal) constraints result in smaller sizes and shorter developmental times for many ectotherms at higher latitudes/altitudes [58,59,60,61] and also in local cold pockets [5,17].

**Figure 13:**
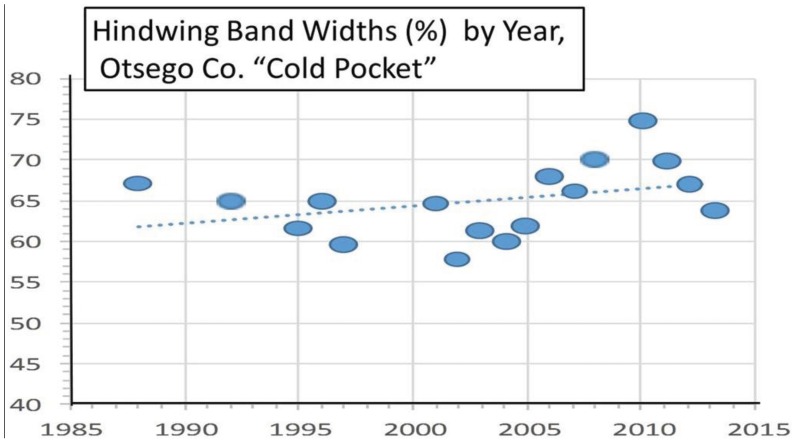
Female hindwing black band widths for the Otsego “cold pocket” as a function of year, showing a slight (non-significant) increase in the trendline. A decrease in band width would be expected if introgression from *P. glaucus* had occurred during this period.

The major collection sites we have sampled for 25–30 years have not shown much variation in mean adult female sizes, however, some locations at the 1–2 generation “voltinism transition zone” (Figure 1), with hybrid interaction and genetic introgression [4,27] have been shown to harbor late-flight (July) homoploid recombinant hybrids that reflect diagnostic traits of both parental species (the univoltine northern *P*. c. and the bivoltine *P*. g.). The forewing lengths of both these early and delayed flight hybrid zone populations of *Papilio* have remained different but steady from 1999–2012. While at the same latitude (43° N) the Battenkill populations of EF(Pc) and LF hybrids are significantly different in size (48 mm *vs.* 51 mm average; Table 1), which illustrates the potential introgression-influenced variation for the “voltinism/size/D-day” model (see Figure 1). A similar increase in forewing sizes of late flight (July) females compared to the early flight *P. canadensis* across the State of Massachusetts (from 1986–1990) showed mean forwewings to be larger for the July (55.0 ± 3.2 mm, n = 26 females) *vs*. May–June (47.3 ± 2.6 mm, n = 15 females). The areas where late flights were reported in Massachusetts are shown (Figure 13, where dotted lines delineate the thermal landscape areas just north of the historical hybrid zone). Clearly, the 2010 landscape (inset in Figure 13) recently suggests a capability for the bivoltine potential to move northward extensively across most of Massachusetts, with likely introgression of various traits from *P. glaucus* [1].

However, even without any obvious genetic introgression in northern Michigan and Wisconsin, since 1997, recent and significant increases in forewing sizes of females have occurred in localized “climatic cold pockets” and some adjacent populations of northern Michigan and Wisconsin, presumably due to unique local landscape patterns of warming during the past 15 years. These local “cold pockets” were seen to reflect a significantly greater rate of winter warming that anywhere else east of the Mississippi River (Figure 5). We also found significant recent increases accumulation of summer Degree­day heat units during the past two decades in and nearby these cold pockets (compared to the major latitudinal sites described in Table 1 and Figure 6; e.g., Isabella, Mackinac/Chippewa, Ontonogon and Alaska). We also found rapid wing size increases in local populations of *P. canadensis* at the “cold pocket”of Michigan’s lower peninsula (Otsego Co.; Figure 8) and nearby (Charlevoix Co.,; Figure 9; and Cheboygan Co.,; Figure 10). Similarly, in the upper peninsula “cold pocket” of northern Wisconsin (near Iron Mt. at the eastern edge of the “cold pocket”) experienced significant summer D-day warming and the forewing lengths showed a corresponding increase (Figure 11a,b).

The “thermal depth” of the cold pockets in northern Michigan and northern Wisconsin can be seen (Figure 2 and Figure 3), and the recent warming temperatures at the edge of these cold pockets can also be seen to reflect rapid mean forewing size increases in local females of *P. canadensis* (Figure 8, Figure 9, Figure 10 and Figure 11). The lack of increase in summer Degree-day accumulations at the major sites in the Upper Peninsula (Ontonagon Co.; Stambaugh in Iron Co.; Mackinac/Chippewa Cos.; Figure 4) stand in contrast to the warming near the edge of this cold pocket (Figure 12; Table 1).

### 4.1. All Life Stages are Important When Considering Adaptations to Thermal Constraints

All developmental stages of insects will likely experience thermal constraints as selection pressures determine their success [61,62,63,64]. We have shown that pupae of *P. canadensis* emerge earlier in the spring than *P. glaucus* [29]. Such early starts would be an ecological advantage where thermal constraints exist, such as throughout the entire hybrid zone from Minnesota to New England (Figure 1). Although post-diapause developmental constraints of pupae during winter and in the Spring are not growth related, they are extremely important for seasonal phenology and reproductive isolation of populations and hybrids [27].

However, such pupal development at lower temperatures for early emergers is not without physiological and ecological costs. Voltinism and diapause “strategies” are variable in insects, but nutrient reserves must be carried forward through winter for post-diapause processes of metamorphosis such as adult formation in pupae, flight, and reproduction [64,65,66,67,68]. This means that winter metabolic stresses (warm and cold) can impact survival and adult size [10,69,70].

Another cost incurred by diapausing *P. canadensis* pupae is lowered survival resulting from short term temperature stress extremes (both warm and cold) during mid-winter or Fall [10,68]. Such short-term stress-induced metabolic expenditures and weight loss in diapausing *P. canadensis* pupae (and also recombinant hybrids of the late flight) compared to *P. glaucus* [10,70] are not simply associated with desiccation as may occur in some Lepidoptera [69], since the percent body water in *P. canadensis* pupae was the same for fall, mid-winter, and spring, at 73%–75% [70]. While the Late-Flight hybrids experience large early metabolic expenditures at cooler temperatures, as did *P. canadensis* [10], they, in contrast, do not emerge early. The total degree-days required to complete post-diapause pupal development and adult emergences for LF hybrids were almost twice those needed for EF (Pc) at 14, 18, and 22° C, and more than twice at 26° C (619 D-days for LF males and 725 D-days for females; 289 D-days male EF, 319 for female EF [27]).

With increasing climatic variation globally [71,72,73,74], fluctuating seasonal temperatures (means and extremes) are becoming more common [75,76,77,78], especially in the winter and spring seasons [79,80,81,82]. Snow cover for overwintering pupae will become less predictable and such variation in air temperature will likely have more severe impacts [61,65,66,71]. Increased daily variance in temperatures (high and low) may also have significant or subtle impacts on insect populations even though the mean daily temperature and the degree-day accumulations may otherwise be identical [79]. Variable winter temperatures may suppress metabolic rates in Lepidoptera [21], and this may generate selection for “deeper diapause” intensity seen in lower latitude *Papilio glaucus* populations from Georgia compared to Pennsylvania and Michigan [10]. Similar increased diapause intensity has been seen in Diptera and Orthoptera [11,12].

### 4.2. Growth Rates and Voltinism

The economic damages associated with increased generations of insect pests [79] in agricultural, silvicultural, and human living environments are likely to be very serious [80,81]. Ambient temperatures affect biochemical reaction rates [82] and thus in combination with the nutritional quality of their hosts, largely govern the growth rate potentials of ectotherms, including immature arthropods [22,23]. Recent reviews [83,84] address multivoltine, bivoltine, univoltine, semi-voltine (2 years per generation; as in most arctic Lepidoptera; [85]) and parti-voltine populations (with 3 or more years per generation, e.g., [63]. An increased number of generations with climate warming has been noted in more than 28 species of Heteropterans and other insects [86,87]. Such increases have also been seen in 275 species of aquatic Odonata [88], geometrid moths [89], dragonflies [90], grape berry moths, [91], spruce bark beetles [92,93], and general Lepidoptera [94,95]. In Coleoptera, heat accumulation associated with unusually warm summers caused a shift from the predominant two-year cycle (semi-voltine) to a one-year cycle (univoltine), thereby doubling the rate of increase and spread of populations of beetles [96,97,98,99].

It has frequently been assumed that insect growth rates are at the maximum that is physiologically possible given a specific ambient temperature and specific amount and quality of resource. However, many animals do not grow at their physiological maximum, even with unlimited food of good quality [100,101,102,103]. Thus, slower (“optimal”?) growth may be favored at certain times under certain conditions, depending on various trade-offs between the costs and advantages of growing rapidly [18,103]. For example, reduced resistance to cold stress [104] or disease [105] can occur at fast growth rates. Locally adapted insect populations may also become differentially adapted for fast growth rates with thermal specialization [106,107,108] or host plant specialization [109].

Parmesan *et al.* [48,110] have pointed out that very few studies have analyzed relationships between climate change for long periods of time, or across the entire geographic range of any species. However, some extensive long term insect studies such as ours here, not only include climate, but also include host use patterns, voltinism, morphology, hybrid zones, and local cold pockets [1,50,111,112]. While *P. canadensis* larvae from thermally-stressed Alaskan populations do grow faster compared to populations with relaxed thermal stress in Michigan [3,8], the adult *P. canadensis* females in Alaska also select the plant species for oviposition which have the highest nutritional quality for the fastest larval growth. In contrast, Michigan females, with relaxed thermal constraints, distribute their eggs more widely across different species of host plants of varying degrees of quality for larval growth with lower quality plants possibly providing “enemy­free-space” for avoiding host specific enemies [4,113,114]. The general result of this behavioral rank­order of *Papilio* oviposition preferences are latitudinal gradients in breadth of host use with alternating bands of specialization and generalization that fit with the alternating “constrained” and relaxed patterns of voltinism potential of the area [4,6]. The complex trade-offs affecting adult size may include different host plant preferences by the different broods of the multivoltine *P. glaucus*, and it is feasible that poorer quality hosts (resulting in smaller pupae/adults) are chosen in one of the summer generations “in order to” escape heavy natural enemy pressure that might focus in on larvae using the usual most nutritious host plants as suggested in the “Voltinism-suitability” model [4,6].

### 4.3. Locally Rapid Responses to Climate Change in “Cold Pockets”

In contrast to the generally relaxed thermal constraints of most Michigan *P. canadensis* populations compared to Alaska (both are obligate diapausers and univoltine), Michigan and Wisconsin “cold pockets” represent very localized thermal constraints (similar to Alaska conditions [5,17]). The cold pocket females have traditionally emerged later than those in surrounding areas and, as in Alaska, they pupate early at smaller sizes than general Michigan *P. canadensis* [5,17]. These cold areas have exerted strong natural selection pressures relative to surrounding populations outside cold pockets. The rapid size (physiological/morphological) responses of females in these warming cold pockets may simply represent adaptive plasticity, rather than a genetically-based size increase (Figure 8, Figure 9, Figure 10 and Figure 11). For example, 1992 was an exceptionally cold year (Figure 5; see also [115]), and it is clear that female size in the following 1993 summer was significantly lower at several locations, possibly as a direct result of “thermal time” constraints during the summer growing season (Figure 8b, Figure 9b and Figure 10b).

However, at least three other potential explanations for increased females sizes in cold pockets exist, including, (1) warmer winters (Figure 5) which may require lower metabolic expenditures for diapausing pupae [10] resulting in more biomass to convert into adult tissue, or (2) perhaps warmer springtimes affected post-diapause development and allowing larger adults to result, as shown in both *P. canadensis* and *P. glaucus* [2]. Also, in local climatic cold pockets of Michigan, the females have historically selected ash (*Fraxinus* spp.) leaves as a favorite, because these tender leaves have the highest nutritional quality compared to fully-expanded leaves of other hosts such as cherry, aspen, poplar, birch, and others (due to delayed bud-break and delayed leafing of ash leaves by 3–6 weeks in the coldest parts; [6]). (3) Recent warming may have allowed these cold pocket females to select host plants such as cherry which allow faster growth and larger pupae (however, oviposition preference behavior of adult females here has not been assayed recently; since 1996 [5]). While LF hybrid oviposition preferences appear be controlled by Z-1inked factors [1,116,117,118], local ash host races in thermally-constrained mountains around the Battenkill River basin in Vermont appear to be a result of, rather than a cause of, evolutionary divergence [119].

Overall, the “voltinism-suitability” concept integrates abiotic (especially thermal) as well as biotic factors such as host plant nutritional quality and natural enemies. As Stamp [120] said: “In temperate regions temperature determines the activity of both herbivores and their enemies, with each having different thermal ranges and optima... The limitations imposed by thermal conditions and host plants influence developmental rate and consequently the number of generations per year. How the developmental periods of generations fit into the growing season may affect foraging patterns and adult size... Enemies can force herbivores into microclimates that are sub-optimal in terms of food quality and temperature, which may contribute to reduction in survivorship of the herbivores... We need models that incorporate the effects of temperature, food quality, and predators...”.

While community interactions between herbivorous insects, host plants, natural enemies, and pollinators may be closely integrated in time and space, it is not likely that climate changes will result in concordant, congruent, or concurrent shifts in the composition of such species [121,122,123,124]. Modelling climate change impacts must include both autecological and synecological aspects and we should strive to better understand the relative importance of biotic and abiotic factors [80,113,125,126,127,128,129] across the entire range of the species of interest. Local adaptations, including genetic evolution and phenotypic flexibility [130,131] also need to be encorporated into climate change models and geographic range predictions for insects [1,29,110,127,132,133,134]. Interactions of changes in plant phenology, nutritional quality, and herbivores will also involve precipitation and carbon dioxide increases as well as temperatures [135].

**Figure 14:**
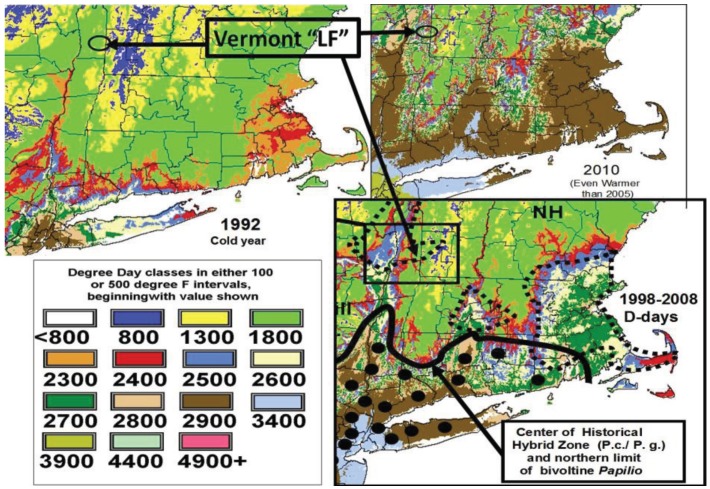
The mean thermal landscape in D-days for NY, PA, MA, and New England during a warm decade (1998–2008) compared to a cold year (1992) and a warm year (2010). The historical hybrid zone (with *P. canadensis* on the northern side) is indicated by the heavy line and D-days indicated by color bands. The dark brown is 2,900–3,400 F and indicates the northern-most limits for bivoltine potential in *P. glaucus* on most plant species. The dotted lines indicate where two generations are not possible and where July (LF *Papilio* hybrids) were seen in the Massachusetts State survey (1986–1990). These “LF” hybrids were larger than the sympatric *P. canadensis* (see Section 4.1) due to recent genetic introgression with correspondingly narrowed hindwing black bands [27,29]. Historical northern limits of dark (mimetic) morph females are shown as dots, and this W(=Y)-linked trait is slow to move even with climate warming [1]. Note the extensive recent warming 2010 (and 2012 was similar) permitting northward movement of the bivoltine potential in eastern Massachusetts, where two generations were basically impossible before 1998.

Increased thermal unit accumulations leading to a potential extra generation at the warmer side of the hybrid zone may enhance northward genetic introgression from the larger, and facultatively diapausing *P. glaucus* [1,136,137,138]. However, multiple matings of these *Papilio* females and males (potentially inter-specifically; [139,140,141]) can make gene flow complicated to assess. Nonetheless, such changes in voltinism and genetic introgression have been shown to impact evolutionary and speciation processes [142,143,144,145,146,147,148,149,150,151], and divergent selection on recombinant hybrids has facilitated such processes in other Lepidoptera [152,153,154]. However, we were nevertheless able to determine that introgression from the southern (larger) *P. glaucus* is unlikely to explain these rapid forewing size increases of female *P. canadensis* locally in cold pockets, since the anal cell hindwing band widths do not get narrower (as in *P. glaucus* and hybrids [29,52,53]) during the same period for Otsego County (Figure 14), and similarly for adjacent Charlevoix and Cheboygan Counties (data not shown). This suggests that phenotypic plasticity in developmental responses (increased size in warming cold pockets) rather than genetic introgression from *P. glaucus* is primarily responsible for these rapid size responses locally in cold pockets.

## 5. Conclusions

Our “Voltinism/Size/Degree-day Model” (Figure 7) explains much of the variance in female sizes across North America better than latitude alone, especially in the mountains of eastern USA. However, depending on the geographic scale and local thermal conditions, these *Papilio* illustrate very different responses to recent climate warming, with no increases in size for any *P. glaucus* populations examined from Michigan to Florida despite major increases in summer thermal accumulations (Figure 2 and Figure 3). The lack of forewing size increases (see Table 1) for the univoltine *P. canadensis* from Alaska to Ontonogon Co. (in the western UP of Michigan) and Mackinac/Chippewa Cos. (in the eastern UP of Michigan) is likely due to the fact that thermal accumulations during summer were not elevated significantly at these sites (Figure 6 and Figure 13; Table 1). In contrast, we report significant female forewing size increases of *P. canadensis* in and near the cold pockets of northern Michigan and northern Wisconsin (during the last 15 years; Figure 8, Figure 9, Figure 10 and Figure 11), and suggest that these larger sizes may in fact have been driven by the significantly warmer summer growing season and associated release from severe thermal constraints on development during the earlier decades of colder summers. The larger body sizes may have also been partially due to the warmer winters and reduced metabolic costs for diapausing and/or post-diapause pupae [10]. These results illustrate the need for sampling populations at different scales. Hybrid introgression has occurred at different rates for different parts of the Z-chromosomes [1,28,151,155,156] and body size increases in the “delayed late flight” recombinant hybrids and the hybrid species (*P. appalachiensis*) likely has a genetic basis [1,9,27,156,157]. However, we show that the rapid size increases of cold pocket *P. canadensis* females with recent warming is more likely to be a result of phenotypic plasticity instead of interspecific genetic introgression.
